# Transcultural Adaptation and Validation of Persian Version of Celiac Disease Questionnaire (CDQ); A Specific Questionnaire to Measure Quality of Life of Iranian Patients

**DOI:** 10.22086/gmj.v0i0.1106

**Published:** 2018-05-19

**Authors:** Farnoush Barzegar, Mohamad Amin Pourhoseingholi, Mohammad Rostami-Nejad, Sepideh Gholizadeh, Mohammad Reza Malekpour, Amir Sadeghi, Kamran Rostami, Iradj Maleki, Shaahin Shahbazi, Mohammad Hassan Emami, Hamid Asadzadeh-Aghdaei, Mohammad Reza Zali

**Affiliations:** ^1^Student Research Committee, Gastroenterology and Liver Diseases Research Center, Shahid Beheshti University of Medical Sciences, Tehran, Iran; ^2^Gastroenterology and Liver Diseases Research Center, Research Institute for Gastroenterology and Liver Diseases, Shahid Beheshti University of Medical Sciences, Tehran, Iran; ^3^Basic and Molecular Epidemiology of Gastrointestinal Disorders Research Center, Research institute for Gastroenterology and 3 Basic and Molecular Epidemiology of Gastrointestinal Disorders Research Center, Research institute for Gastroenterology and; ^4^Department of Gastroenterology, Milton University Hospital, UK; ^5^Gut and Liver Research Center, Mazandaran University of Medical Sciences, Sari, Iran; ^6^Faculty of Medicine, Ilam University of Medical Sciences, Ilam, Iran; ^7^Poursina Hakim Research Institute (PHRI), Isfahan University of Medical Sciences (IUMS), Isfahan, Iran

**Keywords:** Celiac Disease, Quality of Life, Validity, Reliability, Iran

## Abstract

**Background:**

The assessment of health-related quality of life has become an important primary or secondary outcome measure in clinical and epidemiologic studies. The aim of this study was to validate a Persian version of Celiac Disease Questionnaire (CDQ) for Celiac disease (CD) among Iranian patients.

**Materials and Methods:**

The English version of the CDQ adapted to the Persian language by a forward-backward translation by 3 professional bilingual translators (1 medical, 2 nonmedical). The content validity of translated questionnaire were studied by 5 experts who complete the validity form regarding the questionnaire. Then in a pilot study, translated CDQ completed by 81 CD patients who referred to Taleghani Hospital, Tehran. For assessing the validity and reliability of the questionnaire, confirmatory factor analysis and Cronbach’s alpha coefficient have been done, using Lisrel and SPSS software.

**Results:**

Of 81 CD patients entered to this study with mean age of 30.54 years old, 71.6% were female. Also, 56.8% were married and 45.7% were high educated. The mean of CDQ total score was 119.18±34. The calculated Cronbach’s alpha coefficient for CDQ questionnaire was 0.9. Also, for each subgroups the Cronbach’s alpha coefficient were calculated as the following; emotion: 0.92, Social: 0.89, Worries: 0.73, Gastrointestinal: 0.78. Confirmatory factor analysis indicated that all questions could be remain in questionnaire respectively.

**Conclusion:**

The reliability of the Persian version of CDQ was excellent with Cronbach’s alpha coefficients and Persian version of CDQ retains the psychometric properties of the original instrument and should be useful to assess outcome in studies and clinical trials involving Iranian patients with CD.

## Introduction

Celiac disease (CD) was first recognized as a distinct clinical entity more than 60 years ago. However, its full spectrum and impact have only been appreciated over the past decade [1]. CD is an autoimmune inflammatory disorder of the small bowel which affects genetically predisposed individuals upon the ingestion of gluten [2]. CD is characterized by small bowel villous atrophy and lymphocytic infiltration associated with specific antibodies in serum. The clinical spectrum of CD is wide; some patients are asymptomatic with no apparent symptoms, in symptomatic patients, the most common typical presentations are diarrhea, abdominal pain, and weight loss [3] and extra-intestinal manifestations, including iron-deficiency anemia, dyspepsia, osteopor sis, and even infertility or miscarriage are encountered in more than half of CD patients [4].

The primary treatment for CD is life-long elimination of gluten from the diet [5]. Strict gluten-free diet (GFD) allows control of the clinical manifestations and disappearance of disease-specific antibodies from the serum. The benefits of being on GFD are counterbalanced by the burden of GFD[6]. Indeed, a GFD limits preference and also has financial consequences, besides, the gluten-free diet is strict and difficult to accept and follow, leading to modification of eating habits and lifestyle, which changes the patient’s quality of life (QoL) [7]. Patient-reported outcomes (PROs) are increasingly important variables in the evaluation of the impact of chronic diseases and the most commonly used PROs is patient’s perception of health-related QoL (HRQOL) [8]. The assessment of HRQOL has become an important primary or secondary outcome measure in clinical and epidemiologic studies in gastroenterology. However, only limited data are available describing the QoL of patients with CD. Instruments used to measure HRQOL may be generic or disease specific. Generic instruments do not assess specific aspects of a certain disease such as the possible social and financial restrictions due to gluten-free diet (GFD) in CD. Several factors contribute to the negative impact of CD on the HRQOL of affected patients. The chronic nature of CD and the fact that treatment entails a demanding, permanent, restrictive diet with periodic check-ups are foremost among them, but other factors include the limitations that a gluten-free diet imposes on family and social activities, and the psychological distress that all these factors generate. Therefore, the use of both generic and disease-specific HRQOL instruments for clinical studies in gastroenterology is recommended [9]. Recently, a specific HRQOL questionnaire, the ‘‘Celiac Disease Questionnaire’’ (CDQ), was developed for adult patients by Hauser *et al* [9]. The CDQ explores gastrointestinal symptoms, psychological well-being, and social functioning. However, a validated disease-specific HRQOL questionnaire for Iranian patients with CD is still not available. The objective of this study was to develop and validate the Persian cross-cultural adaptation of the CDQ (P-CDQ) for Iranian CD patients and estimate the HRQOL in a representative sample of Iranian adults with CD.

## Materials and Methods

### Target Population

This was a methodological study of translation, validation and cultural adaptation of a HRQOL questionnaire (P-CDQ) for Iranian adult patients with CD. The target population was all of the adults (18 or more years old) CD patients, registered in CD department, Research Institute for Gastroenterology and Liver Diseases Shahid Beheshti University of Medical Sciences, Tehran, Iran. In the confirmatory factor analysis, minimum sample size was determined based on factors rather than variables. For each factor, approximately, 20 samples were required. According to four factors of the questionnaire, 84 Iranian CD patients were selected by convenience sampling. Finally, with three dismissed questionnaires, 81 subjects were analyzed statistically.

### Instrument Development

Disease-Specific HRQOL questionnaire of patients with CD was translated from

English to Persian by 3 celiac experts (1 physician, 2 non physician) and then in a session they met a single translation by assessing of

every questions. In the next step, this Persian

questionnaire was translated to English by several specialists in Gastroenterology and English language. By this mean, differences between English and Pesian versions of the questionnaire was assessed, and by “frequent review process”, decreased to as less as possible.

After the translation, cultural adaption and content validity were assessed and

confirmed by 5 experts who complete the validity form regarding the questionnaire.

The questionnaire had 4 subscales (28 questions), including emotional issues, social problems, disease-related worries, and gastrointestinal symptoms, with 7 items each, inquiring patient’s condition in past two weeks.

Items 2, 3, 6, 10, 14, 16, and 21 stands for emotional issues. Items 4, 9, 15, 18, 20, 22, and 23, stands for social problems. Items 7, 12, 24, 25, 26, 27, and 28, stands for disease-related worries and finally items 1, 5, 8, 11, 13, 17, and 19 constituted gastroi testinal symptoms subscales, respectively.

The study was approved by the Institutional Ethics Committee of the

Research Center for Gastroenterology and Liver Disease (RCGLD), Shahid Beheshti University of Medical Sciences,

Tehran, Iran (IR.SMBU.RIGLD.REC.102).

### Ethical Issue

All patients entered the study, gave their voluntary informed consent after full written explanation of the aims of the study, including considerations regarding ethics and data protection and the anonymous deposition of the questionnaires.

### Statistical Analysis

For assessing the validity and reliability of the questionnaire, confirmatory factor analysis and Cronbach’s alpha coefficient have been done, using Lisrel (Version 8.80, Scientific Software International, Inc.) and SPSS (Version 16, IBM).

## Results

The selected committee of gastroenterologists and other specialists, evaluated all items as relevant to the domains of HRQOL and its content through translation from English to Persian. After adjusting for differences among all translations, the final Persian questionnaire offered to adult CD patients.

In the sample, 28.4% of the subjects were male and 71.6% were female. Mean age of participants was 30.54 years of old and 56.8% of them were married. In terms of education levels, 32.1% were under diploma, 16% had diploma, 45.7% had university certifications, and 6.2% refused to answer. 19.8% of subjects were housekeeper, 25.6% were employee, 3.7% were unemployed, and 46.9% were classified as others. Descriptive properties, including mean and standard deviation of four subscales and the whole questionnaire are shown in Table-1, indicating the total score 119.18 for Disease-Specific HRQOL questionnaire.

**Table-1:** Descriptive Statistics of Subscales Items Of CDQ

**Subscales**	**Mean**	**SD**
**Emotion (E)**	27.64	10.81
**Social (S)**	29.37	10.72
**Worries (W)**	27.11	10.38
**Gastrointestinal (GI)**	35.06	9.76
**Total Score**	119.18	34.00

Cronbach’s alpha coefficient for the whole questionnaire was 0.90 and for emotional issues, social problems, disease-related worries, and gastrointestinal symptoms were 0.92, 0.89, 0.73, and 0.78, respectively (Table-2).

**Table-2:** Internal Consistency of the CDQ

**Subscale**	**Cronbach’s alpha coefficient**	**Items**
**Emotional Issues**	0.92	7
**Social Problems**	0.89	7
**Disease-Related Worries**	0.73	7
**Gastrointestinal Symptoms**	0.78	7
**Whole Questionnaire**	0.90	28

**Figure-1:**
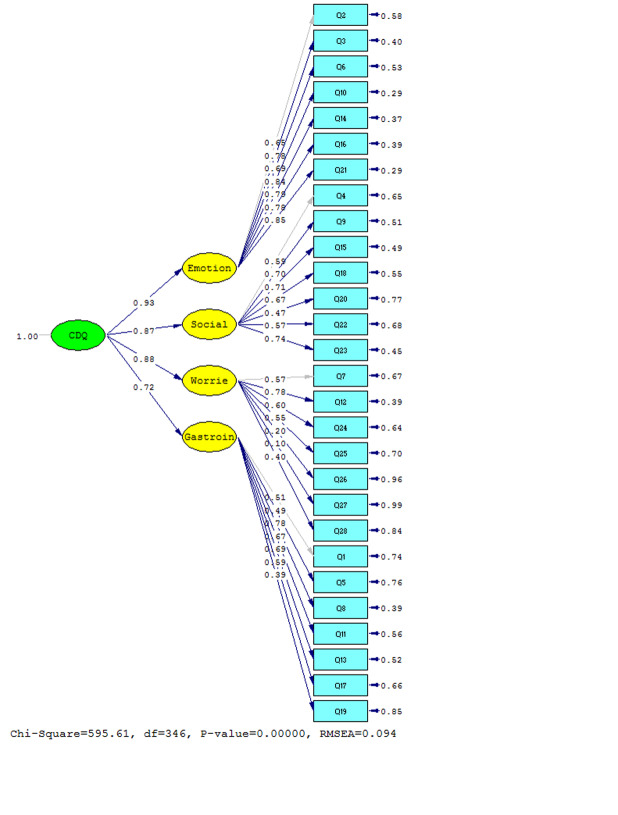
Second-order Factor Analysis for all Questions Included in P-CDQ

**Figure-2:**
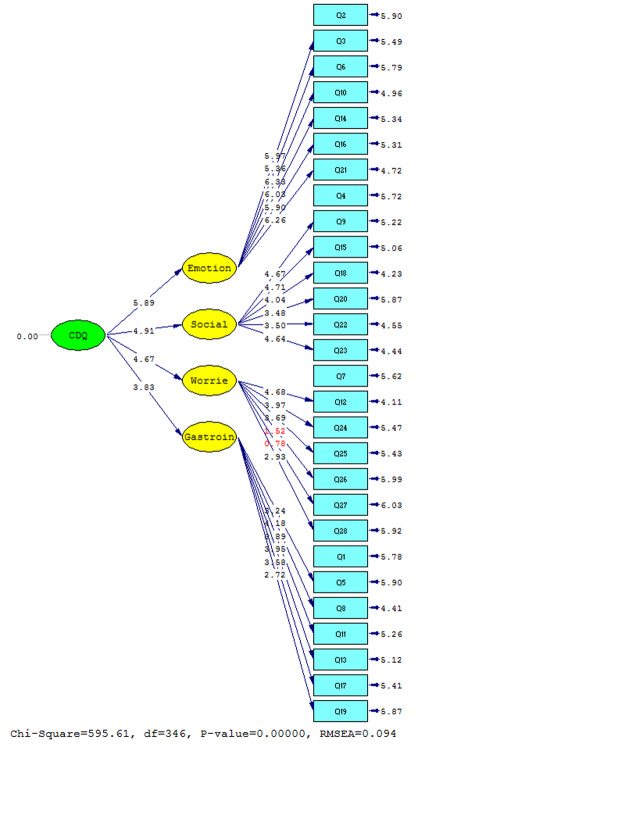
The Acceptable Confirmation of Questions Included in P-CDQ According to Default Factor Analysis

Second-order factor analysis model was fitted to data and the result is shown in Figure-1 and significance of Disease-Specific HRQOL subscales’ coefficients are depicted in Figure-2. According to this model, emotional issues subscale desirably confirmed the first factor analysis with value of 5.89. Also, the values for social problems, disease-related worries, and gastrointestinal symptoms subscales were 4.91, 4.67, and 3.83, respectively which indicated acceptable confirmation of default factor analysis (Figure-2).

Based on T-values (which should not be between -1.96 and +1.96), as a result among the 28 questions in the Table-3, only 2 questions (26 and 27) were rejected.

**Table-3:** Accepting or Rejection Questions According to Factor Analysis Model for P-CDQ

	**Factor Loading**	**Coefficient**	**T-value**	**type**	**Accept / Reject**
**Emotion subscale**	Physically fatigued (Q2)	0.65	-	very desirable	Accept
	Restless (Q3)	0.78	5.97	very desirable	Accept
	Intellectually fatigued (Q6)	0.69	5.36	very desirable	Accept
	Depressed (Q10)	0.84	6.33	very desirable	Accept
	Relaxed (Q14)	0.79	6.03	very desirable	Accept
	Tearful (Q16)	0.78	5.90	very desirable	Accept
	Happy (Q21)	0.85	6.26	very desirable	Accept
**Social subscale**	Invitation/ dinner (Q4)	0.59	-	very desirable	Accept
	Recreation/ Sport (Q9)	0.70	4.67	very desirable	Accept
	Excluded from others (Q15)	0.71	4.71	very desirable	Accept
	Sexual activity (Q18)	0.67	4.04	very desirable	Accept
	(Q20)	0.47	3.48	very desirable	Accept
	Lack of understanding colleagues (Q22)	0.57	3.50	very desirable	Accept
	Professional career (Q23)	0.74	4.64	very desirable	Accept
**Worries subscale**	Inheritance to children (Q7)	0.57	-	very desirable	Accept
	Afraid of getting cancer (Q12)	0.78	4.68	very desirable	Accept
	Expenses /time gluten-free food (Q24)	0.60	3.97	very desirable	Accept
	Problems with health insurance provider (Q25)	0.55	3.69	very desirable	Accept
	Lack of expertise from doctors (Q26)	0.20	1.52	undesirable	Reject
	Diagnosed too late (Q27)	0.10	0.78	undesirable	Reject
	Fear of medical examinations (Q28)	0.40	2.93	very desirable	Accept
**Gastrointestinal subscale**	Sudden urge for bowel movement (Q1)	0.51	-	very desirable	Accept
	Loose bowels (Q5)	0.49	3.24	very desirable	Accept
	Abdominal cramps (Q8)	0.78	4.18	very desirable	Accept
	Bloating (Q11)	0.67	3.89	very desirable	Accept
	Incomplete bowel evacuation (Q13)	0.69	3.95	very desirable	Accept
	Belching (Q17)	0.59	3.58	very desirable	Accept
	Nausea (Q19)	0.39	2.72	very desirable	Accept

However, exclusion of these two questions from structural equation model (MKL and MKL.T Charts) did not cause any notable significant changes according the total model of second-order factor analysis. Therefore inclusion was better.

The considerable point in this model was appropriate fitting model indexes. In accordance with Lisrel output, calculated Chi-Square value was 595.61, with RMSEA=0.094 and P<0.001 (Figure-1 and 2).

## Discussion

According to the results of this methodological study, there were good correlations between the items and their domains, as well as internal consistency. All items showed significant association based on factor analysis, except for question 26 and 27; (Lack of expertise from doctors and diagnosed too late), however the total model showed no significant difference with omitting these two questions. So there could be still including in total Persian CDQ. Also the tool showed good internal consistency according to Cronbach’s alpha coefficient for all dimensions.

CD is the major diagnosable food related disorder, often diagnosed late, presenting with milder and more atypical symptoms [10].

A high prevalence of CD has been found in Iran, in both the general population and the at-risk groups, i.e. patients with type 1

diabetes or irritable bowel syndrome (IBS) [11].

This high prevalence of CD is currently considered the result of a complex interplay between inherent and environmental factors [12].

So, developing a questionnaire to estimate the effect of this chronic disease on QoL of Iranian patients was necessary. The instruments for measuring health-related QoL have been developed in countries with their own languages and based on their cultural characteristics. The CDQ is a valid, reliable and emotional instrument for the assessment of HRQOL for adults CD patients and the translation algorithm, with its several steps, allowed producing validated version of CDQ in other languages and cultures, which sounded natural and easy to understand.

A same Italian study showed a good validated version of the CDQ for Italian patients with good data quality [13]

as well as the French validated version, which showed a rapid instrument to complete and was well accepted by patients with CD [14] and Spanish

version also confirmed the previous studies [15].

Besides, the Turkish version of CDQ which proved to be a reliable and valid instrument for measuring HRQOL, indicated the homogeneity of the items and also the internal consistency to be satisfactory for the original four dimensions of the CDQ by confirmatory factor analysis which is similar to our results for P-CDQ [16].

The advantages of having specific instruments to mea¬sure HRQL based on internationally validated questionnaire is to allow international comparison of the results of national studies. So, transcultural comparisons of adult patients with CD can be conducted using this questionnaire to assess the effects of different culture, life style, environments, etc. on the QoL of this chronic disease.

## Conclusion

The P-CDQ proved a valid and reliable instrument for measuring HRQOL for Iranian patients with CD, in future

cross-sectional, cohort of clinical trial studies.

## Conflict of Interest

The authors declared that there is no conflict of interest
